# Clinic Atlanta Medical College

**Published:** 1874-03

**Authors:** 


### Clinic Atlanta Medical College.

### SURGICAL.

*W. F. Westmoreland, M.D., Professor of Surgery.*

E. A. COBLEIGH, Reporter.

*January* 8.—Case No. 41.—Alice W.; white; aged four years; talipes varus; treated by division subcutaneously of the tendo-achillis, and plantar fascia, and application of club-foot shoe.

Case No. 42.—David G. W.; white; aged eighteen months; brother of last case; talipes varus both feet; treated by section of the tendon in both feet, and the plantar fascia in one; shoes applied.

*January* 10.—Case No. 43.—Lewis T.; white; aged twenty-three; moulder; urethral stricture; treated by introduction of tunneled catheter over filiform bougie; the divulsor was then passed, rupturing the stricture, when No. 18 catheter passed with ease.

*January* 24.—Case No. 47.—John M.; colored; aged forty-five; blacksmith; gonorrhœa:

| | |
|---|---|
| ℞.—Plumbi Acetatis . . . . . | ʒ ss. |
| Zinci Sulphatis . . . . . | gr. xv. |
| Tinct. Opii . . . . . . | f. ʒ iss. |
| Tinct. Catechu . . . . . | f. ʒ ii. |
| Aquæ Puræ . . . . . . | f. ℥ viii. |

M. Sig. Inject a desertspoonful into the urethra thrice daily, after urinating. He will take fifteen drops of the oil of cubebs, three times daily, and gradually increase the dose until a purgative effect is induced; then drop the dose to ten drops, and continue.

Case No. 48.—Ben. B.; colored; aged thirty; merchant; gonorrhœa six months ago; inguinal glands enlarged and indurated; tumor on left side of neck, which has existed for two years. Diagnosis: Sub-acute abscess of cervical gland, which was opened, and half degenerated lymph, with broken down glandular tissue, escaped freely.

Case No. 49.—Tom C.; colored; aged seventeen; sick for a year; tumor just below the angle of scapula, and a similar one in front to right of sternum; both contain fluid; dullness of the right side chest on percussion; no respiratory murmur at the base of the right lung, and no bronchial souffle. Diagnosis: Effusion into pleural sac from inflammation. No treatment, but ordered to return, when paracentesis thoracis will be performed.

*January* 28.—Case No. 50.—Fulton D.; white; aged nineteen; laborer; ulcer of two years standing on radial side of left forearm, evidently syphilitic in character:

| | |
|---|---|
| ℞.—Potassii Iodidi . . . . . . | ℥ iss. |
| Tinct. Gentianæ . . . . . | f. ℥ ii. |
| Tinct. Stillingiæ . . . . . | f. ℥ ii. |
| Aquæ . . . . . . . . | f. ℥ iv. |

M. Sig. Tablespoonful three times daily, in a pint of water.

### EYE AND EAR.

*A. W. Calhoun, M.D., Professor of Diseases of the Eye and Ear.*

*January* 14.—Case No. 18.—Caroline C.; æt. thirty-eight years. This woman is suffering from granular lids, which were treated by applications of—

| | |
|---|---|
| ℞.—Argenti Nitratis . . . . . . | grs. x. |
| Aquæ Puræ . . . . . | f. ℥ j. |

to diseased parts with camel's hair brush, once daily.

*January* 17.—Case No. 19.—William W.; æt. sixty-nine years; white. This man came before the class suffering from same complaint as the preceding patient; the same treatment was ordered for this case as for the other.

*January* 21.—Case No. 20.—Charley R.; æt. thirteen years; white. Has congenital cataract of both eyes. These were treated by decission, and patient is doing nicely.

Case No. 21.—Terrell R.; brother of last patient; five years of age. Came before class January 21st, with congenital cataract of both eyes. Treated by same plan as preceding case.

*January* 28.—Case No. 22.—Evelina B.; forty-eight years old; washerwoman. Patient says that six years ago left eye became very much inflamed, and itched terribly. One year ago she lost her sight altogether in that eye, and the other eye became affected. Adhesions between the iris and capsule of lens were easily distinguished by casual examination. The diagnosis in this case was secondary cataract. Dr. Calhoun proceeded to operate for extraction of the diseased lens, but, before commencing, informed patient that probably the operation would not restore sight to any degree, but would lessen her liability to the constantly recurring and troublesome iritis to which she was subject. After extraction of lens she could count the fingers when held up before her, and distinguish one from the other.

